# Exploring the Kinetic and Thermodynamic Relationship of Charge Transfer Reactions Used in Localized Electrodeposition and Patterning in a Scanning Bipolar Cell

**DOI:** 10.3389/fchem.2019.00340

**Published:** 2019-05-14

**Authors:** Trevor M. Braun, Daniel T. Schwartz

**Affiliations:** ^1^Functional Nanostructured Materials Group, Materials Science and Engineering Division, National Institute of Standards and Technology, Gaithersburg, MD, United States; ^2^Electrochemical Materials and Interfaces Laboratory, Chemical Engineering Department, University of Washington, Seattle, WA, United States

**Keywords:** bipolar electrochemistry, electrodeposition, material fabrication, current distribution, electrolyte design

## Abstract

Bipolar electrochemistry involves spatial separation of charge balanced reduction and oxidation reactions on an electrically floating electrode, a result of intricate coupling of the work piece with the ohmic drop in the electrochemical cell and to the thermodynamics and kinetics of the respective bipolar reactions. When paired with a rastering microjet electrode, in a scanning bipolar cell (SBC), local electrodeposition and patterning of metals beneath the microjet can be realized without direct electrical connections to the workpiece. Here, we expand on prior research detailing electrolyte design guidelines for electrodeposition and patterning with the SBC, focusing on the relationship between kinetics and thermodynamics of the respective bipolar reactions. The kinetic reversibility or irreversibility of the desired deposition reaction influences the range of possible effective bipolar counter reactions. For kinetically irreversible deposition systems (i.e., nickel), a wider thermodynamic window is available for selection of the counter reaction. For kinetically reversible systems (i.e., copper or silver) that can be easily etched, tight thermodynamic windows with a small downhill driving force for spontaneous reduction are required to prevent metal patterns from electrochemical dissolution. Furthermore, additives used for the bipolar counter reaction can influence not only the kinetics of deposition, but also the morphology and microstructure of the deposit. Cyclic voltammetry measurements help elucidate secondary parasitic reduction reactions occurring during bipolar nickel deposition and describe the thermodynamic relationship of both irreversible and reversible bipolar couples. Finally, finite element method simulations explore the influence of bipolar electrode area on current efficiency and connect experimental observations of pattern etching to thermodynamic and kinetic relationships.

## Introduction

Bipolar electrochemistry—a phenomenon involving spatially segregated, equal and opposite reduction and oxidation charge transfer reactions on an electrically floating electrode—has recently proven valuable for a range of applications where traditional electrochemical methods are inadequate. Bipolar electrochemical reduction and oxidation reactions are driven by the potential gradient in solution that polarizes an electrically floating electrode (aka bipolar electrode or BPE) positioned within the electric field. When the solution ohmic resistance responsible for generating potential gradients is substantial relative to the charge transfer resistance of these reactions, a portion of the total applied current can pass through the BPE, manifesting in spatially bifurcated reduction and oxidation reactions on a single conductor. Because charge must be conserved on the BPE, the reduction current equals that of oxidation. Complete understanding of the intricate coupling between the kinetics of the bipolar redox reactions, their thermodynamic relationship, and ionic/electronic transport through the cell is critical to designing effective bipolar electrochemical systems.

Precise control of both reduction and oxidation reactions on an electrode free of direct electrical contact has generated several new bipolar electrochemical applications, ranging from electroanalytical chemistry to material fabrication. A powerful development involves addressing large microfabricated electrode arrays with a single set of feeder electrodes, demonstrating high throughput screening of material properties (Munktell et al., 2015), measuring electrocatalytic activity coupled to electrochemiluminescence signatures (Chow et al., 2009; Lin et al., 2012; Xiao et al., 2017), and developing sensors based on metal dissolution (Chow et al., 2010; Fosdick et al., 2013). The potential gradient driving electrochemistry in bipolar systems has further been utilized to develop compositionally graded material systems (Ulrich et al., 2008; Ishiguro et al., 2011; Tisserant et al., 2015; Xu et al., 2018). Bipolar electrochemistry is also useful in device fabrication, including deposition of non-line-of-sight interconnects between electrically isolated posts (Bradley et al., 1997, 1999), production of anisotropic functionalized microparticles (Loget et al., 2012; Tiewcharoen et al., 2017), and growth of single metal nanowires (Wood and Zhang, 2015).

Previous work by our group demonstrated that a rastering microjet nozzle can be employed for localized bipolar electrodeposition and patterning on an electrically floating substrate, a system we called a scanning bipolar cell (SBC) (Braun and Schwartz, 2015, 2016a,b,c). Initial applications of the SBC on a copper bipolar electrode involved copper electrodeposition in the region beneath the nozzle (near-field) and copper dissolution of the substrate material in the region surrounding the nozzle (far-field) (Braun and Schwartz, 2015). The equal but opposite nature of bipolar electrochemistry resulted in a “sculpting” of the originally planar substrate; in a high faradaic efficiency system like copper, every copper ion reduced beneath the nozzle resulted in an atom of copper metal etched in the far-field. Reduction of other metal cations, such as Ni^2+^, on a copper BPE resulted in a similar displacement of copper in the far-field while nickel electrodeposited beneath the nozzle (Braun and Schwartz, 2016b). In these experiments the far-field area for oxidation was about 1,000 × greater than the near-field reduction region, resulting in only a nanometer of material etched for every micron of material deposited. Controlling the initial quantity of copper charge on the substrate available for oxidation translated to self-limited patterning with the SBC, where local nickel deposition (electron acceptor) terminated upon complete etching of the copper “fuel” (electron donor). Rather than rely on metal dissolution as the bipolar counter reaction, an electrolyte-born redox couple was used to generalize electrodeposition in the SBC for a diverse range of metals (Braun and Schwartz, 2016c). Computational methods validated analytical scaling relationships approximating current flow and coupling between thermodynamics, kinetics, and transport in the SBC (Braun and Schwartz, 2016a).

Electrochemical advanced manufacturing methods have experienced a recent growth similar to traditional additive manufacturing techniques (i.e., stereolithography, fused deposition modeling, selective laser sintering, etc.) as industry shifts to more sustainable fabrication options (Braun and Schwartz, 2016d; Hirt et al., 2017). In particular, advantages of electrodeposition in additive manufacturing include improved material flexibility (capabilities include metals, alloys, semiconductors, and polymers) while achieving superior voxel resolutions, far below that of state-of-the-art two-photon stereolithography (Kawata et al., 2001). Standard scanning ion conductance microscopy (SICM) pipettes have fabricated features <500 nm in size (Momotenko et al., 2016), while electrohydrodynamic printing has shown material deposition rates on the order of 1–10 μm/s (Hirt et al., 2017). Bipolar electrochemistry with the SBC offers the unique ability to do electrodeposition-based additive manufacturing without requiring electrical connections to the workpiece. This attribute is particularly useful when fabricating on complex substrates at sub-micron lengthscales, such as in electronics manufacturing, where electrically connecting to patterned conducting and non-conducting surfaces can be challenging.

The work presented here expands upon prior experimental and computational research with the SBC. Characteristics of kinetically irreversible and reversible electrodeposition chemistries are described in more detail, including electroanalytical measurements of mixed potential systems comprised of representative bipolar couples. Patterned arrays of nickel demonstrate the challenges with parasitic reduction chemistries and the impact of bipolar additives on deposit appearance. Finite element method simulations are used to assess the impact of the far-field couple's kinetics on current efficiency, a circuit element that becomes increasingly relevant as substrate geometry and aspect ratio are constrained. Finally, simulations describe the coupling between applied current and spatiotemporal deposit stability for reversible electrolyte systems, correlating theory to experimental observations.

## Methods

### Scanning Bipolar Cell

**Figure 1:**
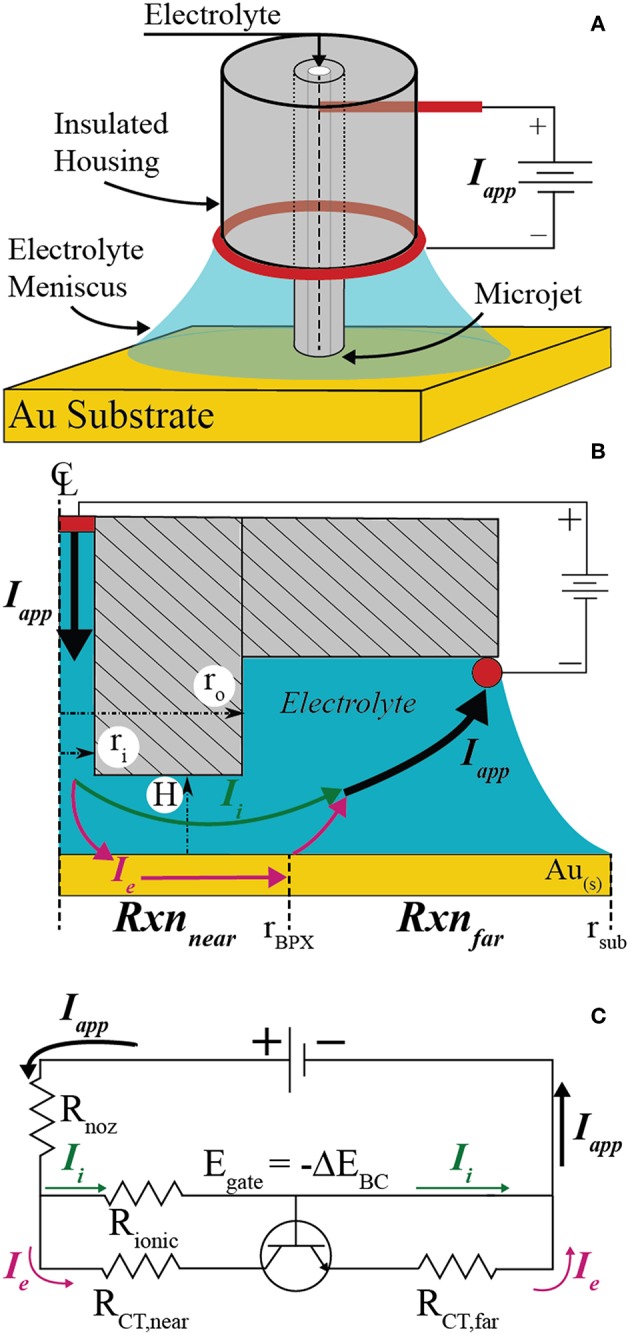
**(A)** Schematic of the scanning bipolar cell (SBC) and components. **(B)** To-scale axisymmetric geometry used in finite element method simulations when *H* = *2r*_*i*_ with key features and current pathways emphasized. **(C)** Equivalent circuit approximating current flow pathways through the SBC.

Figure 1A shows a perspective view of the key features and configuration of the electrochemical cell used to perform localized bipolar electrodeposition on electrically floating conductive substrates. Electrolyte is pumped through an electrically insulating microcapillary nozzle with a syringe pump. A platinum wire “feeder” electrode (the anode when configured to drive local reduction electrochemistry) is inserted in the microcapillary upstream of the nozzle outlet, where electrolyte jets onto the conductive substrate (bipolar electrode, or BPE). The electrolytes forms a liquid pool on the BPE, contacting a platinum “feeder” ring electrode (i.e., cathode when performing local reduction electrochemistry) attached to the acrylic housing of the microjet nozzle. The microjet nozzle dimensions and flowrates are such that considerable mass-transport is achieved, with limiting current densities exceeding 10 A/cm^2^ in some cases (Nelson and Schwartz, 2005).

Figure 1B depicts the axisymmetric geometry and key features of the SBC used in finite element simulations for the case where *H* = *2r*_*i*_. The inner (*r*_*i*_ = 100 μm) and outer (*r*_*o*_ = 335 μm) radii and the fly-height of the nozzle above the substrate (*H*) tailor the ohmic drop in the annular gap between the nozzle and substrate, controlling the breakdown of ionic and electronic current pathways in the electrochemical cell. Current sourced from the upstream platinum feeder electrode (*I*_*app*_) has two pathway options upon exiting the nozzle: (1) it can travel entirely through the electrolyte to the downstream ring feeder electrode entirely as ionic current (*I*_*i*_), or, (2) it can undergo charge transfer at the substrate in the region beneath the nozzle, pass as electronic current (*I*_*e*_) through the BPE, undergo a second (equal and opposite) charge transfer reaction in the far-field area, and then travel as ionic current to the feeder ring electrode. The fraction of applied current that passes through the electronic pathway (*I*_*e*_) defines the bipolar current efficiency (*BCE*)

(1) $$\begin{array}{l} BCE=\frac{I_{e}}{I_{app}} \end{array}$$

and is coupled to the ohmic drop through solution, charge transfer kinetics of the bipolar reactions, and thermodynamic relationship of the bipolar pair.

The current pathways depicted in Figure 1B are approximated by the equivalent circuit in Figure 1C. The resistance for current flow through the ionic pathway is dominated by the ohmic resistance beneath the microjet nozzle (*R*_*ionic*_). The total resistance to current flow through the conductive substrate is the sum of the charge transfer resistances related to the kinetics of the bipolar reactions beneath the nozzle (*R*_*CT,near*_) and in the far-field (*R*_*CT,far*_), assuming the electrical resistance of the conductor is negligible. When the bipolar reaction pair is thermodynamically uphill (i.e., non-spontaneous), current cannot flow through the conductor until the potential drop through solution exceeds the thermodynamic potential difference of the bipolar couple (Δ*E*_*BC*_)

(2) $$\begin{array}{l} \Delta E_{BC}=E_{red}^{eq}-E_{ox}^{eq} \end{array}$$

where the subscripts *red* and *ox* refer to the bipolar reduction and oxidation reactions occurring on the substrate. Equation 2 effectively acts as a threshold voltage. The equivalent circuit models this as an ideal transistor having infinite resistance until the gate voltage (*E*_*gate*_ = -Δ*E*_*BC*_) is exceeded, after which the transistor has zero resistance and current flows at a rate regulated by other circuit elements. The transition between *off* and *on* states is considered to be instantaneous. Thus, current flows entirely through the ionic pathway (*off*) until the potential drop through the annular gap (*E* = *I*_*app*_*R*_*ionic*_) is > –Δ*E*_*BC*_. When the potential drop exceeds *E*_*gate*_ (*on*), current may flow through the electronic branch of the parallel circuit at a rate regulated by the relationship between ohmic and kinetic resistances. Previous studies described *R*_*ionic*−_ using primary current simulations and explored the relationship between ohmic and kinetic resistance (*R*_*CT,near*_) for a reversible bipolar couple (i.e., Δ*E*_*BC*_ = 0) and for a thermodynamically uphill bipolar couple (Δ*E*_*BC*_ < 0) (Braun and Schwartz, 2016a). In those simulations the kinetic resistances of the far-field reactions were negligible, arising from the experimental far-field region having a much larger surface area than the near-field region and thus lower current densities.

### Chemicals

Unless noted otherwise, the following chemicals were used as received: NiSO_4_·5H_2_O (Sigma Aldrich, 99–102.0%), L-ascorbic acid (Sigma Aldrich, 99%), CuSO_4_·5H_2_O (Fisher Scientific, technical grade), AgNO_3_ (Sigma Aldrich, 99.0%), FeSO_4_·7H_2_O (Sigma Aldrich, 98%), Fe_2_(SO_4_)_3_·5H_2_O (Sigma Aldrich, 97%), K_2_SO_4_ (Alfa Aesar, 98.0%), concentrated HNO_3_ (Fisher Scientific, certified ACS plus), and concentrated H-_2_SO_4_ (Mallinckrodt Chemicals, 95–98%). All aqueous electrolytes were prepared with high purity deionized (DI) water.

### Bipolar Plating Electrolyte

Local bipolar electrodeposition in the SBC is experimentally demonstrated for two characteristic systems: kinetically irreversible (Ni) and kinetically reversible (Cu or Ag) electrodeposition chemistries. Prior work outlined the electrolyte design guidelines necessary to achieve both spatially and temporally stable deposits using the SBC for these bipolar systems (Braun and Schwartz, 2016c). Specifically, kinetically irreversible electrodeposition chemistries can be paired with any bipolar oxidation chemistry resulting in Δ*E*_*BC*_ < 0, whereas kinetically reversible electrodeposition chemistries must be paired with a bipolar oxidation reaction that produces a marginally downhill thermodynamic relationship (i.e., Δ*E*_*BC*_ > 0). These metastable formulations for reversible bipolar systems are similar to electroless deposition systems, except with only a 10–50 mV of downhill driving force. This results in a thermodynamic buffer for the desired oxidation chemistry to occur at lower overpotentials than metal etching, as the rastering of the SBC nozzle exposes previously deposited metal to electrochemically oxidizing conditions. Because irreversible deposition systems are kinetically passivated against dissolution, a wider range of bipolar oxidation couples can be utilized.

For kinetically irreversible nickel electrodeposition, ascorbic acid (AA) oxidation forming dehydroascorbic acid (DHAA) and protons is the bipolar counter reaction

$$\begin{array}{lll} Near & : & \;Ni^{2+}+{2e}^{-}\to Ni_{\left(s\right)},\;E_{near}^{eq}=-0.29V\\ Far & : & \;AA\to DHAA+{2H}^{+}+{2e}^{-},\;E_{far}^{eq}=0.30\;V\;[\text{Scheme }1]\\ Net & : & \;Ni^{2+}+AA\to Ni_{\left(s\right)}+DHAA+{2H}^{+},\;\Delta E_{BC}=-0.59V \end{array}$$

producing a stable electrolyte with a negative Δ*E*_*BC*_ in 0.1 mol/L NiSO_4_ + 0.01 mol/L ascorbic acid (pH = 2.9). Equilibrium potentials are calculated by the Nernst equation using the above reactant concentrations. All potentials reported are referenced to the standard hydrogen electrode (SHE) unless otherwise noted. Formation of a passivating oxide layer protects nickel from bulk electrooxidation during SBC operation, allowing design of thermodynamically uphill electrolytes that require applied current to drive the bipolar reaction sequence in Scheme 1.

For kinetically reversible copper electrodeposition, ascorbic acid (AA) oxidation is also selected as the bipolar counter reaction:

$$\begin{array}{lll} Near & : & \;Cu^{2+}+{2e}^{-}\to Cu_{\left(s\right)},\;E_{near}^{eq}=0.30\;V\\ Far & : & \;AA\to DHAA+{2H}^{+}+{2e}^{-},\;E_{far}^{eq}=0.29\;V\;[\text{Scheme }2]\\ Net & : & \;Cu^{2+}+AA\to Cu_{\left(s\right)}+DHAA+{2H}^{+},\;\Delta E_{BC}=+0.01V \end{array}$$

The above estimated equilibrium reduction potentials for 0.05 mol/L CuSO_4_ + 0.005 mol/L ascorbic acid (pH = 2.6) produce a metastable electrolyte with a marginally positive Δ*E*_*BC*_. This downhill thermodynamic driving force provides protection for previously deposited copper but is not sufficient to drive heterogeneous deposition, let alone overcome the nucleation barrier for homogenous reduction of cupric ions in solution.

For kinetically reversible silver electrodeposition, ferrous (Fe^2+^) ion oxidation to ferric (Fe^3+^) ion acts as the bipolar counter reaction:

$$\begin{array}{lll} Near & : & \;Ag^{+}+e^{-}\to Ag_{\left(s\right)},\;E_{near}^{eq}=0.67V\\ Far & : & \;Fe^{2+}\to Fe^{3+}+e^{-},\;E_{far}^{eq}=0.63\;V\;[\text{Scheme }3]\\ Net & : & \;Ag^{+}+Fe^{2+}\to Ag_{\left(s\right)}+Fe^{3+},\;\Delta E_{BC}=+0.04V \end{array}$$

The equilibrium potentials above are estimated from open circuit potential measurements in a solution containing 0.01 mol/L AgNO_3_ + 0.1 mol/L K_2_SO_4_ and a solution containing 0.01 mol/L FeSO_4_ + 0.005 mol/L Fe_2_(SO_4_)_3_ + 0.1 mol/L K_2_SO_4_. Measured open circuit potentials varied slightly, falling between 0.67 and 0.68 V for Ag(I)/Ag_(s)_ and between 0.63 and 0.66 V for Fe(III)/Fe(II). Similar to Scheme 2, this ≈ +40 mV downhill driving force is less than the nucleation overpotential for homogeneous reduction of silver, providing a thermodynamic window for Fe^2+^ oxidation to occur before electrochemical silver dissolution in bipolar experiments. However, long-term stability (>1 day) of this electrolyte has not been evaluated. As noted previously (Braun and Schwartz, 2016c), inclusion of both ferrous sulfate and ferric sulfate in the electrolyte is required to tailor the equilibrium potential. As a result, Fe^3+^ reduction to Fe^2+^ competes with the desired silver electrodeposition beneath the nozzle, reducing the overall faradaic efficiency of the bipolar system.

### Bipolar Electrode Substrate Preparation

All electrodeposition experiments were performed on gold substrates. Silicon wafers with 50 nm gold on a 5 nm titanium adhesion layer were prepared using an E-beam evaporator at the University of Washington Nanofabrication Facility. Prior to bipolar experiments, the gold substrates were cleaned via 20 cyclic voltammetry sweeps at 50 mV s^−1^ from −0.25 to 1.5 V vs. SCE in a 1 mol/L H_2_SO_4_ solution with a Pine Model AFRDE5 Bipotentiostat. Clean gold substrates were then rinsed with deionized (DI) water and dried with N_2_ gas. Substrates were masked to make a circular exposed area of 0.45 cm^2^. For the electrolytes flow rates and small number of printed features used here, the meniscus was pinned on the SBC feeder cathode/outer nozzle housing. As the droplet of pooled electrolyte grew during liquid injection, it drained over the much larger masked substrate. All electrodeposition steps were carried out at room temperature.

### Electroanalytical Measurements

Cyclic voltammetry (CV) was performed on a Biologic model VSP potentiostat with a platinum wire counter electrode and saturated mercury sulfate (SSE) reference electrode. All potentials have been referenced to the SHE unless otherwise noted. A gold Pine rotating disk electrode (RDE) with a 0.5 cm diameter was used for all RDE experiments. Between experiments involving nickel, the Au RDE was polished with 4000 grit SiC and rinsed in deionized water. Between experiments involving silver or copper, the Au RDE was rinsed in 1 mol/L HNO_3_ and subsequently rinsed in deionized water.

Cyclic voltammetry exploring nickel deposition in Scheme 1 used 0.1 mol/L NiSO_4_ while varying ascorbic acid concentration from 0 to 0.2 mol/L. Potential sweeps were done at 20 mV/s beginning at −0.4 V vs. SSE, sweeping to −1.6 V vs. SSE, and then to 0.5 V vs. SSE for 5 cycles without rotation. The 1st cycle for each concentration was selected for comparison. Cyclic voltammetry exploring silver deposition in Scheme 3 compared three solutions: (1) 0.01 mol/L FeSO_4_ + 0.005 mol/L Fe_2_(SO_4_)_3_ + 0.1 mol/L K_2_SO_4_, (2) 0.01 mol/L AgNO_3_ + 0.1 mol/L K_2_SO_4_, and (3) 0.01 mol/L AgNO_3_ + 0.01 mol/L FeSO_4_ + 0.005 mol/L Fe_2_(SO_4_)_3_ + 0.1 mol/L K_2_SO_4_. Potential sweeps were done at 20 mV/s beginning at 0.1 V vs. SSE, sweeping to −0.2 V vs. SSE, and then to 0.4 V vs. SSE for 10 cycles while rotating at 0 RPM or 100 RPM (1 RPM = 0.105 rad/s). The potential limits for CV of solution (1) containing only iron components varied slightly, by beginning at 0 V vs. SSE and sweeping negative to −0.3 V. Cycles 2–10 were selected for comparison of electrolyte components from Scheme 3.

Linear sweep voltammetry (LSV) on 25 μm diameter Au microlectrodes (UMEs) was also used to evaluate the nickel deposition system. Prior to voltammetric measurements, the Au UMEs were polished on 4000 grit SiC, rinsed in deionized water, rinsed in 1 mol/L HNO_3_, and rinsed again in deionized water. The same nickel electrolyte used in RDE measurements was also used on UMEs. Voltammetry in electrolytes without nickel in solution used 0.1 mol/L K_2_SO_4_ as supporting salt. Potential sweeps were done at 20 mV/s beginning at −0.4 V vs. SSE and sweeping to −1.9 V vs. SSE. Voltammetry on microelectrodes exploring the impact of pH in the absence of ascorbic acid used electrolytes with 0.1 mol/L NiSO_4_ and dosed in H_2_SO_4_ to vary pH.

### Characterization Tools

An Olympus BX51 optical microscope with an Olympus QColor3 digital camera using 5.0x objectives was used to take optical micrographs of each sample. Coarse and grainy silver deposits were visibly metallic, however, appeared dark in coloration when imaged with bright-field on the optical microscope. Therefore, silver optical micrographs were acquired using dark-field imaging. An Oakton model no. 510 pH/conductivity meter was used to measure pH.

### Computational Methods

Finite element method computations were performed in the axisymmetric 2D computational domain shown in Figure 1B to assess the current flow pathways in the SBC. All simulations use a microjet nozzle with dimensions of *r*_*i*_ = 100 μm and *r*_*o*_ = 335 μm, consistent with experiments, unless otherwise noted. Details on the computational methods, including relevant equations and boundary conditions, may be found in the Appendix.

Simulations for near-field copper reduction on a gold substrate with ascorbic acid as the far-field bipolar counter reaction in Scheme 2 were used to explore electrochemical behavior for a kinetically reversible bipolar electrodeposition reaction. The Cu^2+^/Cu_(s)_ redox couple is kinetically reversible, using kinetic parameters taken from literature (Mattsson and Bockris, 1959): *i*_*o,Cu*_ = 33.5 A/m^2^, α_*a,Cu*_ = 0.73, and *n* = 2. The ascorbic acid redox couple is also kinetically reversible, however, only trace amounts of dehydroascorbic acid are present in solution. We deal with this uncontrolled, trace dehydroascorbic acid in a manner that is easy to implement, produces results that are consistent with experimental measurables (such as open-circuit potential, threshold voltages/currents, etc.), and whose magnitude (within reasonable bounds) has negligible influence on the computational results we report. Specifically, for the electrode boundary conditions (Appendix), we use the Butler-Volmer form with *f*_*AA*_ = 1 for the oxidation branch and set a small limiting current for dehydroascorbic acid reduction (*i*_*L*_ = *i*_*o,AA*_) with *g*_*AA*_ = (1-*i/i*_*o,AA*__−_). Kinetic parameters for ascorbic acid are taken from literature (Tanaka and Tamamushi, 1964): *i*_*o,AA*_ = 10.2 A/m^2^, α_*a,AA*_ = 0.20, and *n* = 2.

Simulations exploring the impact of substrate area on BCE assume irreversible deposition beneath the nozzle, employing Tafel kinetics for reduction and using a Δ*E*_*BC*_ = −0.1 V. The generic far-field oxidation chemistry is assumed to have kinetics similar to that of ascorbic acid. Kinetic parameters for the irreversible reduction chemistry are: *i*_*o,near*_ = 10 A/m^2^, α_*near*_ = 0.50, and *n* = 2. Kinetic parameters for the oxidation bipolar couple are: *i*_*o,far*_ = 1 A/m^2^ or 10 A/m^2^, α_*far*_ = 0.50, and *n* = 2. Parameters are varied to explore how factors such as nozzle dimensions, electrochemical cell configuration, and oxidation kinetics impact the BCE as substrate area (*A*_*subs*_) is constrained. Table 1 presents the parameter combinations used in simulations. The distance of the feeder cathode from nozzle center is *r*_*cathode*_.

**Table 1:** Parameters varied in simulations exploring the impact of substrate area on current flow in the SBC and bipolar current efficiency (BCE) values at select ratios of substrate area to nozzle area.

	***i-_**0, *far***_*** **(A m^**−2**^)**	***r_***i***_*** **(μm)**	***r_***o***_*** ** (μm)**	***r_***cathode***_*** **(mm)**	***BCE* at maximum**	***BCE at A_***Subs***_/A_***noz***_* = 4**	***BCE at A_***Subs***_/A_***noz***_* = 1.44**
Parameter set 1	10	100	335	2	0.921	0.686	0.200
Parameter set 2	1	100	335	2	0.901	0.641	0.142
Parameter set 3	10	200	670	2	0.957	0.756	0.243
Parameter set 4	10	100	335	8	0.951	0.686	0.200
Parameter set 5	10	100	670	2	0.975	0.686	0.200
Parameter set 6	10	50	335	2	0.946	0.611	0.161

**Table 2:** Parameters used in computational models exploring bipolar copper deposition (Scheme 2) and a generic irreversible reduction chemistry used to assess the impact of substrate area on current flow in the SBC.

**PARAMETERS FOR BIPOLAR CU REDUCTION AND AA OXIDATION SIMULATIONS**		
*i_*o,Cu*_*	33.5 A/m^2^	Exchange current density for copper redox couple
*i_*o,AA*_*	10.2 A/m^2^	Exchange current density for ascorbic acid redox couple
*α_*a,Cu*_*	0.73	Transfer coefficient for copper redox couple
*α_*a,AA*_*	0.2	Transfer coefficient for ascorbic acid redox couple
*$E_{Cu}^{eq}$*	0.30 V	Equilibrium potential for copper redox couple
*$E_{Cu}^{eq}$*	0.29 V	Equilibrium potential for ascorbic acid redox couple
*H*	15 μm	Fly-height of nozzle
*I_*app*_*	20 or 200 μA	Applied current to cell
κ	1 S/m	Conductivity of electrolyte
**PARAMETERS FOR SIMULATIONS ON IMPACT OF SUBSTRATE AREA**		
*i_*o,near*_*	10 A/m^2^	Exchange current density for local redox couple
*i_*o,far*_*	1 or 10 A/m^2^	Exchange current density for far-field redox couple
*α_*a,near*_*	0.5	Transfer coefficient for local redox couple
*α_*a,far*_*	0.5	Transfer coefficient for far-field redox couple
*ΔE_*BC*_*	−0.1 V	Equilibrium potential difference of bipolar redox pairs
*H*	10 μm	Fly-height of nozzle
*I_*app*_*	100 μA	Applied current to cell
κ	1 S/m	Conductivity of electrolyte

Mesh refinement in the regions of high potential gradient was used so that the overall charge balance and the substrate integral on the bipolar electrode both converged to <0.01% error. This resulted in about 200,000 to 500,000 mesh elements and computation times ranging from 1 to 10 min for a typical converged solution. All simulations were performed on a Dell Optiplex 980 desktop computer with an Intel Core i5 CPU@ 3.20 GHz and 8 GB RAM using Windows 7 Enterprise 64-bit operating system. FEM simulations employed the secondary current distribution module in COMSOL version 5.3.

## Results and Discussion

Bipolar electrochemical systems require careful mating of the thermodynamics and kinetics of the bipolar reaction couple, a result of the potential-induced bifurcation of the substrate into separated reduction and oxidation regions. In the case of a rastering electrode (i.e., SBC microjet nozzle), the local reduction and oxidation regions move with the nozzle, exposing previously deposited material (in cathodic regions) to anodic environments capable of etching the material. How the deposited material responds to the oxidizing environment depends on whether it is passive or active at the potentials of the oxidizing region.

**Figure 2:**
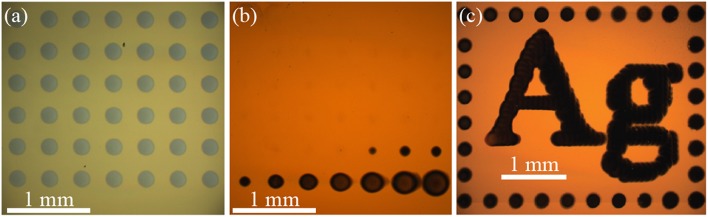
Optical micrographs of metal patterns deposited from a SBC with a 200 μm ID nozzle at 400 μL/min flowrate. **(a)** A 7 × 7 array of nickel grown in 0.1 mol/L NiSO_4_ + 0.2 mol/L ascorbic acid at 900 μA with 4,500 μC per deposit, a nozzle fly-height of 18 μm, and 400 μm deposit spacing. **(b)** A 7 × 7 array of silver grown in 0.05 mol/L AgNO-_3_ at 50 μA with 250 μC per deposit, a nozzle fly-height of 30 μm, and 400 μm deposit spacing. **(c)** Pattern consisting of 282 individual silver deposits grown in 0.01 mol/L AgNO_3_ + 0.01 mol/L FeSO_4_ + 0.005 mol/L Fe_2_(SO_4_)_3_ at 15 μA with 30 μC per deposit, a nozzle fly-height of 30 μm, and 75 μm deposit spacing.

Figure 2a shows an optical micrograph of an array of nickel material electrodeposited with the SBC in a 0.1 mol/L NiSO_4_ + 0.2 mol/L ascorbic acid electrolyte (Δ*E*_*BC*_ = −0.59 V). Each deposit in the 7 × 7 array is grown at *I*_*app*_ = 900 μA, *Q* = 4,500 μC, and *H* = 18 μm. Deposit spacing is 400 μm and electrolyte flowrate is 400 μL/min. The same spacing and flowrate are used for all experiments unless otherwise noted. Individual deposits in the array appear optically identical, as they were deposited at the same conditions. Despite nickel oxidation being thermodynamically preferential to ascorbic acid oxidation ($E_{Ni}^{eq}$ = −0.29 V and $E_{AA}^{eq}$ = 0.30 V), formation of a thin passivating oxide layer after deposition kinetically prevents electrochemical etching of the material as the SBC moves across the substrate and the deposits experience an oxidizing potential.

Several metal deposition systems do not have the irreversibility characteristic of nickel passivation, and can dissolve in anodic environments. For example, Figure 2b shows an optical micrograph of an array of silver deposits, a kinetically reversible deposition chemistry, where most of the deposited material has etched from the substrate. The electrolyte contains only 0.05 mol/L AgNO_3_ with the bipolar counter reaction being water oxidation

(3) $$\begin{array}{l} Far:\;2H_{2}O\to O_{2\left(g\right)}+{4H}^{+}4e^{-},\;E_{far}^{eq}\approx 1.02\;V \end{array}$$

resulting in a bipolar couple with a Δ*E*_*BC*_ = −0.30 V. Each deposit in this 7 × 7 array was grown at *I*_*app*_ = 50 μA, *Q* = 250 μC and *H* = 30 μm. Like nickel in Figure 2a, silver oxidation is thermodynamically preferential to water oxidation ($E_{Ag}^{eq}$ = 0.72 V and $E_{H2O}^{eq}$ = 1.02 V). In contrast to nickel, oxidation of silver is kinetically active and will electrochemically etch when exposed to anodic potentials in the far-field during SBC operation. The result is an array where the material deposited earliest, and exposed to oxidation longest, has been fully removed from the substrate (the SBC scanned from left to right, top to bottom in the Figure 2 experiments). History of silver etching is evident in the systematic decrease of deposit size over the final 10 deposits. In stationary bipolar electrochemical systems separation of the cathodic and anodic regions remain fixed, providing inherent stability for electrodeposited metals. With the SBC, moving anodic and cathodic regions during patterning requires temporal deposit stability to be linked to the thermodynamics of the bipolar pair.

As described in the experimental methods section, temporal deposit stability of kinetically reversible materials systems can be achieved by selecting a bipolar counter reaction producing a marginally positive Δ*E*_*BC*_. Figure 2c shows an optical micrograph of a silver pattern containing 282 individual silver deposits on a gold substrate that is stable over 20 min during fabrication [adapted from (Braun and Schwartz, 2016c)]. The bipolar counter reaction is oxidation of ferrous ion (Fe^2+^) to ferric ion (Fe^3+^) by Scheme 3 in an electrolyte containing 0.01 mol/L AgNO_3_ + 0.01 mol/L FeSO_4_ + 0.005 mol/L Fe_2_(SO_4_)_3_. Each deposit is grown at *I*_*app*_ = 15 μA, *Q* = 30 μC and *H* = 30 μm. Deposit spacing is 75 μm. In contrast to Figure 2b, Fe^2+^ oxidation is thermodynamically preferential to Ag^+^ oxidation ($E_{Ag}^{eq}$ = 0.67 V and $E_{Fe\left(II\right)}^{eq}$ = 0.63 V). The downhill thermodynamics (Δ*E*_*BC*_ = +40 mV) of this bipolar couple protects silver from electrochemical dissolution, but is modest enough to prevent spontaneous reduction of silver in solution. In both types of kinetic systems applied current drives local deposition on the substrate. For silver, only a small amount of current (15 μA in Figure 2c) is needed to drive heterogeneous nucleation on the substrate. Nickel requires a much larger applied current (900 μA in Figure 2a) to overcome the thermodynamic barrier and drive the bipolar reactions. The applied current for driving nickel deposition could be reduced by selecting a bipolar counter reaction producing a less negative Δ*E*_*BC*_. In the following sections, electrolyte design attributes for characteristic irreversible and reversible bipolar electrodeposition systems will be discussed in more detail.

### Irreversible Materials Systems

**Figure 3:**
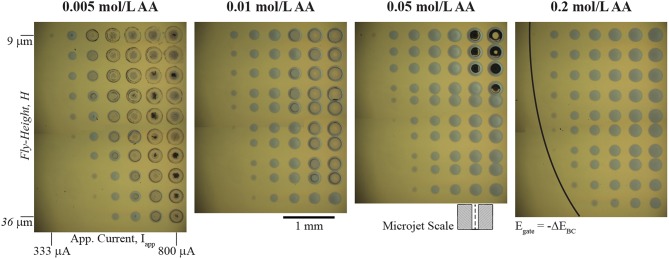
Optical micrographs of arrays of nickel deposits in 0.1 mol/L NiSO_4_ at the indicated ascorbic acid concentrations. 10 × 10 arrays of nickel deposits vary applied current (*I*_*app*_) from 100 to 800 μA (lowest 3 columns not shown) and nozzle fly-height (*H*) from 9 to 36 μm. Each deposit has a 5 s dwell time. A 200 μm ID nozzle at 400 μL/min electrolyte flowrate and deposit spacing of 400 μm was used for each experiment. Certain rows in the 0.01, 0.05, and 0.2 mol/L ascorbic acid arrays are more closely spaced than others due to a misstep in the y-axis motor during movement. The schematic of the microjet nozzle is provided for scale. The black line in the 0.2 mol/L array indicates the threshold potential (Δ*E*_*BC*_) to drive bipolar nickel deposition and ascorbic acid oxidation.

A wide range of redox couples for the bipolar counter reaction are available for local electrodeposition of kinetically irreversible material systems in the SBC. Essentially, any redox couple with an equilibrium potential more positive than that of the metal ion reduction chemistry can be utilized, with the consequence that bipolar couples with more negative values of Δ*E*_*BC*_ require more applied current to drive both reactions. Selecting an appropriate bipolar counter reactant also requires consideration of that constituent's impact on the characteristics of the electrodeposited metal and the reaction itself. Solution additives can affect electrodeposition in several ways, including: significantly altering deposit morphology; metal ion chelation, which changes the reversible potential of metal deposition; and controlling the pH, which can impact the stability of the metal phase in solution and influence secondary parasitic reactions (Schlesinger, 2010). Thus, it is important to consider how the ascorbic acid additive serving as the bipolar counter reactant affects electrodeposition of nickel beyond bipolar behavior. Figure 3 shows a series of optical micrographs depicting 10 × 10 nickel arrays in 0.1 mol/L NiSO_4_ at varying ascorbic acid concentrations. In each array, the applied current (*I*_*app*_) is varied from 100 to 800 μA (the lowest 3 columns not shown have no nickel deposited) and nozzle fly-height (*H*) is varied from 9 μm to 36 μm. Each deposit was grown using a 5 s dwell time. The ohmic resistance beneath the nozzle (*R*_*ionic*_) and near-field charge transfer resistance (*R*_*CT,near*_*)* depicted in Figure 1C dictate current flow in the SBC. Prior simulations (Braun and Schwartz, 2016a) of the primary current distribution in the SBC show that *R-*_*ionic*_ is related to the electrolyte conductivity (κ) and nozzle geometry by

(4) $$\begin{array}{l} R_{ionic}=\frac{1}{4\kappa r_{i}}\left(1-\frac{2}{\pi \sqrt{1+0.16{\left(\frac{H}{r_{i}}\right)}^{2}}}\right)+\frac{Ln\left(\frac{r_{o}}{r_{i}}\right)}{2\pi \kappa H}. \end{array}$$

As fly-height is increased, the ionic resistance defined by Equation 4 decreases, and less of the applied current follows the bipolar pathway resulting in smaller nickel deposits in the Figure 3 arrays. Secondary current distribution simulations (Braun and Schwartz, 2016a) for nickel indicated that the kinetic resistance of the local reaction at high overpotentials beneath the nozzle scales by

(5) $$\begin{array}{l} R_{CT,near}=\frac{RT}{\left(I_{app}-I_{\min}\right)\alpha_{j}n_{j}F} \end{array}$$

*I*_*min*_ is the minimum current required to polarize the substrate sufficiently (i.e., exceed -Δ*E*_*BC*_) that drives both nickel reduction beneath the nozzle and ascorbic acid across the far-field. The portion of the arrays without nickel deposits have yet to exceed the threshold substrate polarization. Once the minimum current for –Δ*E*_*BC*_ has been exceeded, additional applied current beyond *I*_*min*_ can participate in bipolar nickel deposition. Increasing applied current reduces the kinetic resistance by Equation 5, driving more current through the bipolar pathway resulting in larger nickel deposits. Of course, larger deposits at higher currents are also partially due to an increase in total charge passed during the 5 s dwell time of each deposit.

Before the kinetic pathway is activated (i.e., substrate polarization exceeds –Δ*E*_*BC*_) all of the applied current passes through the ionic pathway and the potential drop through the gap (*E*) follows

(6) $$\begin{array}{l} E=I_{app}R_{ionic.} \end{array}$$

Once *E* exceeds –Δ*E*_*BC*_, current can flow through the bipolar electrode via charge transfer reactions and nickel deposits appear on the substrate. The deposits appearing at the lowest current for each fly-height (where *E* ≈ –*E*_*BC*_) can be used to estimate *R*_*ionic*_. For example, in the 333 μA column of the 0.05 mol/L AA solution the faintest trace of nickel material is visible at a fly-height of 15 μm. With a Δ*E*_*BC*_ of −0.56 V, Equation 6 gives an estimation for *R*_*ionic*_ as 1,680 Ω. Similarly, the faintest deposit in the *I*_*app*_ column of 411 μA appears at *H* = 27 μm, giving a value for *R*_*ionic*_ of 1,360 Ω. However, estimations for *R*_*ionic*_ using the analytical expression in Equation 4 for these fly-heights are 14,000 Ω and 8,200 Ω, respectively. This large deviation from theoretical expectation likely reflects the non-idealized geometry in the experimental microjet nozzles, which have rounded edges and asymmetries as a result of hand-polishing of the glass capillaries. However, the expected qualitative trends are reproduced in the Figure 3 arrays.

A section of the nickel arrays in Figure 3 show crater-like deposits at the conditions of highest applied current and lowest fly-height. The quantity of these features decreases with increasing ascorbic acid concentration, suggesting they are related to the ascorbic acid reactant. Since the craters appear at conditions of greatest substrate polarization (high *I*_*app*_ and low *H*), they are most likely caused by a secondary reduction reaction occurring at potentials more negative than nickel deposition. Deposition of iron group metals (Ni, Co, Fe) from aqueous electrolytes is often accompanied by proton and water reduction reactions

(7) $$\begin{array}{l} 2H^{+}+2e^{-}\to H_{2} \end{array}$$

(8) $$\begin{array}{l} {2H}_{2}O+{2e}^{-}\to H_{2}+OH^{-}. \end{array}$$

**Figure 4:**
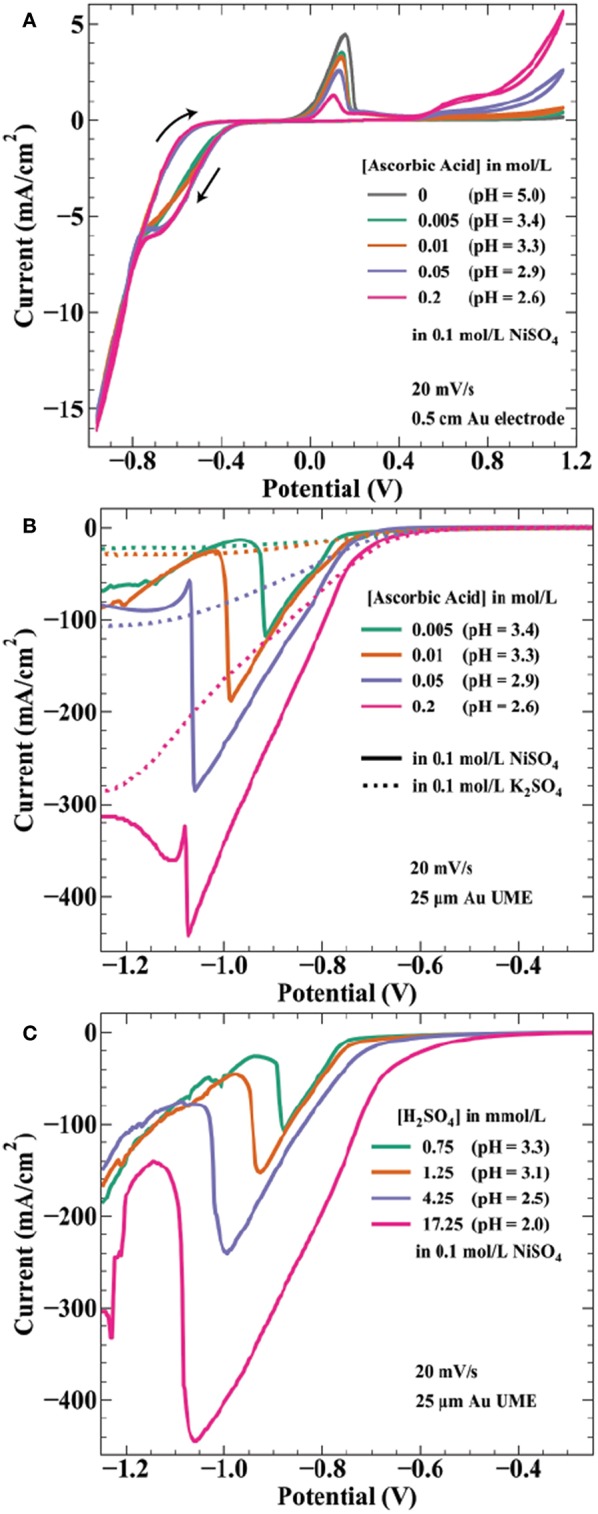
Voltammetric measurements of nickel deposition in ascorbic acid and sulfuric acid containing electrolytes. (**A)** Cyclic voltammetry at 20 mV/s on a 0.5 cm diameter Au electrode in 0.1 mol/L NiSO_4_ and the indicated ascorbic acid concentrations. Voltammograms initiated at 0.24 V, swept negative to −0.96 V, positive to 1.14 V, ending at 0.24 V. Linear sweep voltammetry at 20 mV/s from 0.24 to −1.26 V on a 25 μm diameter Au microelectrode for **(B)** the indicated ascorbic acid concentrations in 0.1 mol/L NiSO_4_ (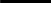) or 0.1 mol/L K_2_SO_4_ (….) and for **(C)** the indicated H_2_SO_4_ concentrations in 0.1 mol/L NiSO_4_. pH values are indicated for each solution.

Ascorbic acid is a weak acid with two protons (pK_a_ values of 4.2 and 11.6) and concentration changes affect the solution pH. The measured pH values of the solutions used in Figure 3 are 3.4, 3.3, 2.9, and 2.6 by increasing ascorbic acid concentration. Thus, onset potentials for both proton reduction and water reduction will vary between each array. Figure 4A shows cyclic voltammetry of the electrolytes used in Figure 3 experiments. As potential is swept negative, current is first observed around −0.26 V. The onset potential for this wave (taken at a current density of −0.1 mA/cm^2^) shifts from −0.25 to −0.28 V as ascorbic acid increases from 0.005 to 0.2 mol/L. This potential range is in agreement with estimations by the Nernst equation for proton reduction of −0.15 to −0.20 V for pH = 3.3–2.6 solutions, with additional overpotential likely due to sluggish kinetics of proton reduction on gold (Norskov et al., 2005).

The estimated Nernst potential for Ni^2+^ reduction of −0.28 V suggests that nickel deposits concurrently with proton reduction at potentials negative of this value. Near −0.75 V, the individual curves from each concentration merge to the same path, possibly indicating a shift to current dominated by nickel deposition. The CV shows hysteresis on the return sweep between −0.8 and −0.4 V, likely a result of the 20 mV/s scan rate. As the potential sweeps positive the characteristic nickel passivation behavior is observed: increasing anodic current due to nickel dissolution followed by a sharp decrease in current resulting from formation of a passive hydroxide layer and subsequent thin oxide (Smith et al., 1987; Scherer et al., 2003). The onset of nickel dissolution (taken at a current density of 0.1 mA/cm^2^) shifts more positive with increasing ascorbic acid concentration (and proton reduction), occurring at −0.054 V for 0.005 mol/L AA and −0.006 V for 0.2 mol/L AA. This positive 48 mV shift correlates well with the 59 mV/pH unit (47 mV from pH = 3.4 to pH = 2.6) expected for electrochemical formation of NiO (Sato and Okamoto, 1963). Interestingly, the presence of additional ascorbic acid results in decreased peak current prior to metal passivation. This effect is counter to the expectation of a decrease in pH, which should make formation of a passivating hydroxide layer more difficult. At still higher potentials, ascorbic acid oxidation to dehydroascorbic acid and protons initiates around 0.4 V with higher current densities achieved for more concentrated AA solutions. This onset potential is consistent with literature values of the reversible potential of ascorbic acid in this pH range (Borsook and Keighley, 1933).

Due to the high convective transport of the jetted electrolyte, current densities achieved in the SBC can often exceed 100 mA/cm^2^. For example, at a fly-height of 15 μm with an *I-*_*min*_ of 333 μA, the total current available for nickel reduction at *I*_*app*_ = 566 μA is 233 μA. In reality, a portion of this current passes through the ionic pathway (Figure 1C) as shunt current. Even assuming a BCE of 50%, the current density at this condition (scaled by the deposit area with a diameter = 250 μm) is 237 mA/cm^2^. As a result, voltammetry using microelectrodes was utilized to better connect electroanalytical measurements to transport conditions and lengthscales relevant to SBC operation. Linear sweep voltammetry (LSV) on a 25 μm gold microelectrode (UME) seen in Figure 4B was further used to probe the potential regime where proton and water reduction compete with nickel deposition. Potential is swept from −0.4 to −1.9 V at 20 mV/s. Ascorbic acid variation in 0.1 mol/L K_2_SO_4_ (dashed lines) shows proton reduction followed by water reduction in the absence of nickel deposition. Similar to CVs in Figure 4A, current from proton reduction initiates prior to nickel deposition. For example, in 0.2 mol/L ascorbic acid + 0.1 mol/L NiSO_4_ current in the voltammogram begins to deviate from the nickel free electrolyte at about −0.75 V, indicating the onset of nickel reduction at a more negative potential. This ≈ 450 mV overpotential ($E_{Ni}^{eq}$ = −0.28 V) before nickel reduction current is observed is due to a combination of nucleation overpotential and nickel reduction having very slow kinetics (Tanaka and Tamamushi, 1964). A decrease in the faradaic efficiency for nickel deposition is observed with additional proton in solution; estimations at −0.86 V being 76, 74, 55, and 46% with increasing ascorbic acid concentration.

A previous hypothesis for the craters depicted in Figure 3 was coevolution of hydrogen gas bubbles disrupting nickel nucleation on the gold bipolar electrode (Braun and Schwartz, 2016c). This now seems unlikely, as the voltammetry shows proton reduction occurs at potentials more positive than that of nickel deposition. At potentials more negative than nickel reduction, significant hydroxide generation from water reduction can cause metal-hydroxide phases to form in the deposit. To counter this, buffered electrolytes are often employed to combat local pH changes near the electrode interface (Ji et al., 1995; Zech and Landolt, 2000). As potential is swept further negative in Figure 4B, a sharp decrease in current is observed for the nickel-containing electrolytes. A previous study on deposition of Ni, Co, and Fe from an aqueous solution demonstrated quenching of metal film growth concurrently with the onset of OH^−^ generation by water reduction, producing a hydroxylated surface (i.e., ${\text{Ni}(\text{OH})}_{x}^{2-x}$) that blocks subsequent metal deposition (Wang et al., 2016). The sharp decrease in current observed in Figure 4B is similarly attributed to formation of a nickel hydroxide surface phase at the onset of water reduction, terminating nickel deposition. This voltammetric feature shifts to more negative potentials as ascorbic acid (and proton) concentration increases, by ≈ 200 mV/pH unit over the range explored. The Nernst potential shift by pH is greater than expected for water reduction, 59 mV/pH unit, suggesting ascorbic acid also has buffering qualities similar to boric acid. Indeed, voltammetry in ascorbic acid free electrolyte when pH is controlled by additions of sulfuric acid (Figure 4C) shows a potential shift of the spike by only 122 mV/pH. This value is comparable to a similar study observing a voltammetric spike attributed to OH^−^ generation by Equation 8, reporting a 110 mV/pH unit shift of the voltammetric feature (Ritzert and Moffat, 2016). Both potential shifts of the spike exceed that expected by thermodynamics for the 2 electron water reduction reaction, possibly indicating a more complicated mechanism for formation of the nickel hydroxide surface species than simply

(9) $$\begin{array}{l} Ni^{2+}+xOH^{-}\to {Ni\left(OH\right)}_{x}^{2-x} \end{array}$$

where hydroxide is primarily produced by water reduction.

The electroanalytical measurements in Figure 4 help clarify the formation of crater-like deposits observed in Figure 3. At sufficiently negative cathodic overpotentials (i.e., high *I*_*app*_ and low *H*), water reduction occurs subsequently with nickel deposition, producing OH^−^ and causing nickel deposition to terminate due to formation of a nickel hydroxide surface phase. The local solution potential is at a minimum (most reducing) directly beneath the nozzle, increasing in potential (less reducing) radially from the nozzle center in a gaussian-like profile. As a result, the craters are composed of a thin nickel hydroxide surface phase in the high overpotential region beneath the center of the nozzle, with subsequent passed charge only manifesting in water reduction. Further from the nozzle center, reduction of Ni^2+^ at more positive potentials forms a ring of thicker nickel metal. The black material in a few of the crater-like deposits is likely residue from bulk Ni(OH)_2_ precipitation after sustained OH^−^ generation during water reduction. Figure 4B indicated that the overpotential necessary to drive OH^−^ generation is greater for higher ascorbic acid concentration due to its buffering qualities as well as a decrease in pH. Thus, fewer crater-like deposits appear in Figure 3 for higher ascorbic acid concentrations.

**Figure 5:**
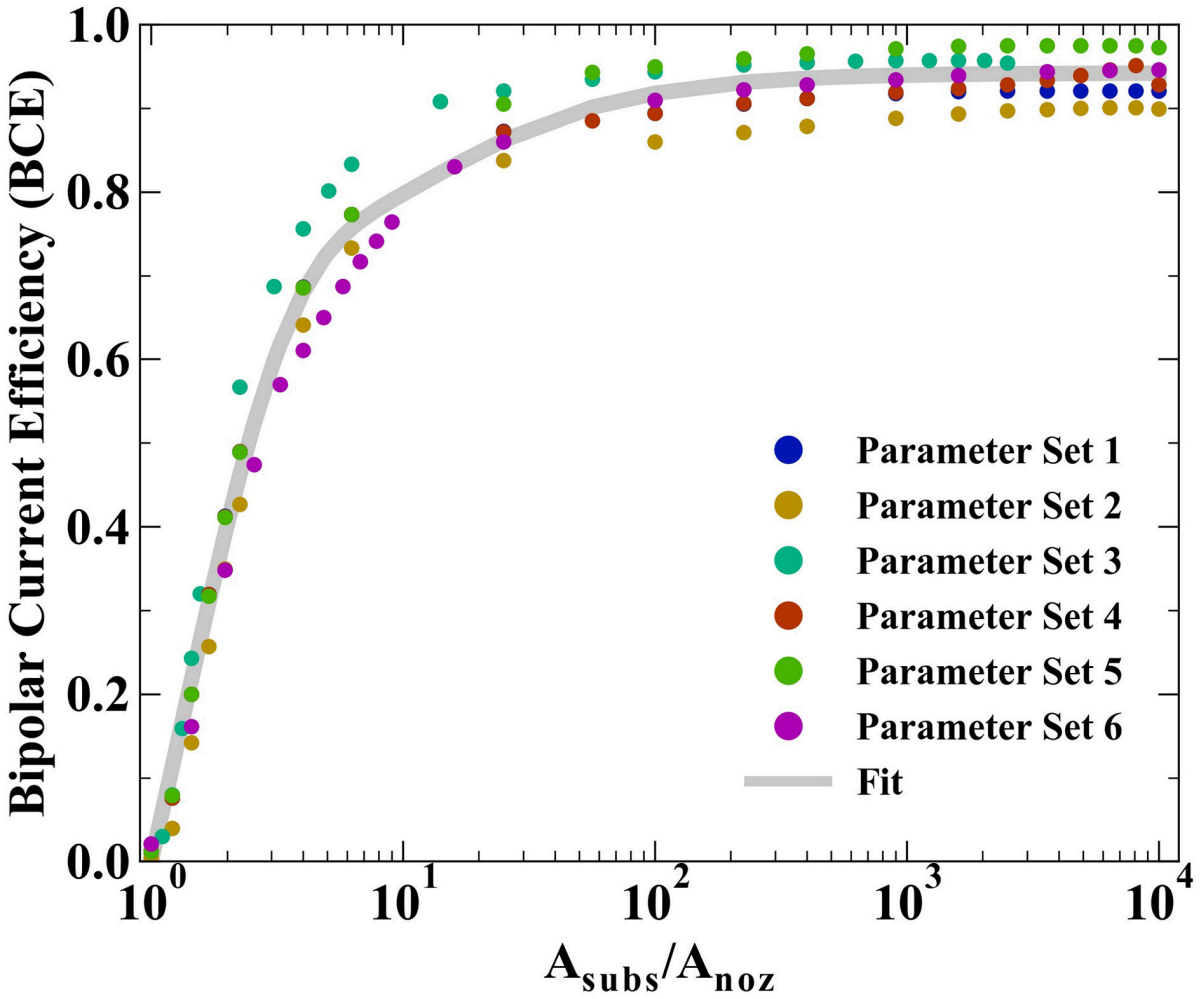
Bipolar current efficiencies (BCE) plotted vs. the ratio of total substrate area (*A*_*subs*_) to nozzle area (*A*_*noz*_) defined by *r*_*i*_. The varied parameters are highlighted in Table 1 and the fit is modeled by Equation 10.

All prior experimental systems using the SBC have been designed such that the total charge transfer resistance of the far-field oxidation chemistry is negligible relative to that of the near-field reduction reaction, a result of the much larger area of the far-field region. Factors influencing the kinetic resistances in the equivalent circuit in Figure 1C are the exchange current densities (*i*_*o,i*_) and transfer coefficients (α_*a,i*_) for the bipolar reactions, as well as the total area each reaction is taking place in. Typical SBC experiments use a 200 μm inner diameter nozzle (the approximate dimension of a metal deposit) on a masked substrate with 0.45 cm^2^ circular area, resulting in an oxidation area roughly 1000 × larger than the reduction area. Here, finite element method computations are used to explore how reducing the substrate area available for oxidation impacts the *BCE* of the SBC. Six different kinetic and geometric parameter combinations are tested (see Table 1), varying either the exchange current density of the far-field reaction (*i*_*o,far*_), the geometry of the microjet nozzle through *r*_*i*_ or *r*_*o*_, or the distance of the feeder cathode from the nozzle center (*r*_*cathode*_). Figure 5 shows the *BCE* plotted vs. the ratio of the total circular substrate area (*A*_*subs*_) to the nozzle area (*A*_*noz*_) calculated by *r*_*i*_. The applied current is 100 μA and fly-height is 10 μm in all simulations. Generally, the effect of reducing the substrate area results in a decay of *BCE* from a plateau to zero as substrate area becomes increasingly constrained. When the area of the substrate is large relative to the SBC nozzle, the *BCE* is at a high value, varying little as *A*_*subs*_/*A*_*noz*_ decreases from 10,000 to 100. A substantial decrease in *BCE* occurs below a ratio of 10, quickly decaying to 0 as the substrate area approaches the same size as the nozzle area. The parameter sets tested in Table 1 show greater variation at values of large *A*_*subs*_/*A*_*noz*_, where changing nozzle geometry is expected to impact *R*_*ionic*_ more than the *R*_*CT,far*_. Changing the cathode location has a minimal impact on *BCE*, with an average of 0.65% difference between parameter sets 1 and 4.

The simulated *BCE* for all 6 parameter sets was fit to a decay equation of the form

(10) $$\begin{array}{l} BCE=A\left(1-e^{\left(-B^{*}(\frac{A_{subs}}{A_{noz}}-1\right)}\right)+{\left(D-A\right)}^{*}\left(e^{\left(-\frac{C}{\left((\frac{A_{subs}}{A_{noz}}\right)}\right)}\right) \end{array}$$

where A, B, and C are constants fit by non-linear least squares regression (equaling 0.744, 0.778, and 15.1, respectively) and D is the average of the maximum values for *BCE* in Table 1. The first term in Equation 10 captures the sharp decay below *A*_*subs*_/*A*_*noz*_ ≈ 10 while the second term captures the more gradual decay in *BCE* at higher ratios of *A*_*subs*_/*A*_*noz*_. Despite the variation in *BCE* across the parameters explored in Table 1, particularly at higher values of *A*_*subs*_/*A*_*noz*_, Equation 10 qualitatively describes how restricting the substrate area affects current flow in the SBC. Simulations suggest that the substrate area should be at least 2.45 × that of the microjet nozzle area to achieve 50% BCE by Equation 10 for a fly-height of 10 μm.

### Reversible Materials Systems

As outlined above, kinetically reversible electrodeposition chemistries such as Cu and Ag require a more thermodynamically favorable bipolar oxidation redox couple (i.e., Δ*E*_*BC*_ > 0) to prevent metal etching as the SBC rasters across the substrate. If the bipolar counter reaction is too reducing (i.e., Δ*E*_*BC*_ >> 0) metal cations will homogenously reduce in solution. Prior studies with the SBC demonstrated temporally stable patterning of kinetically reversible metals by designing electrolytes where Δ*E*_*BC*_ is only marginally positive by a few 10s of millivolts (Braun and Schwartz, 2016c). This provides enough of a thermodynamic cushion that the desired bipolar counter reaction is driven before metal etching during SBC patterning, but not enough driving force to overcome the nucleation barrier to cause spontaneous, homogeneous reduction in solution.

**Figure 6:**
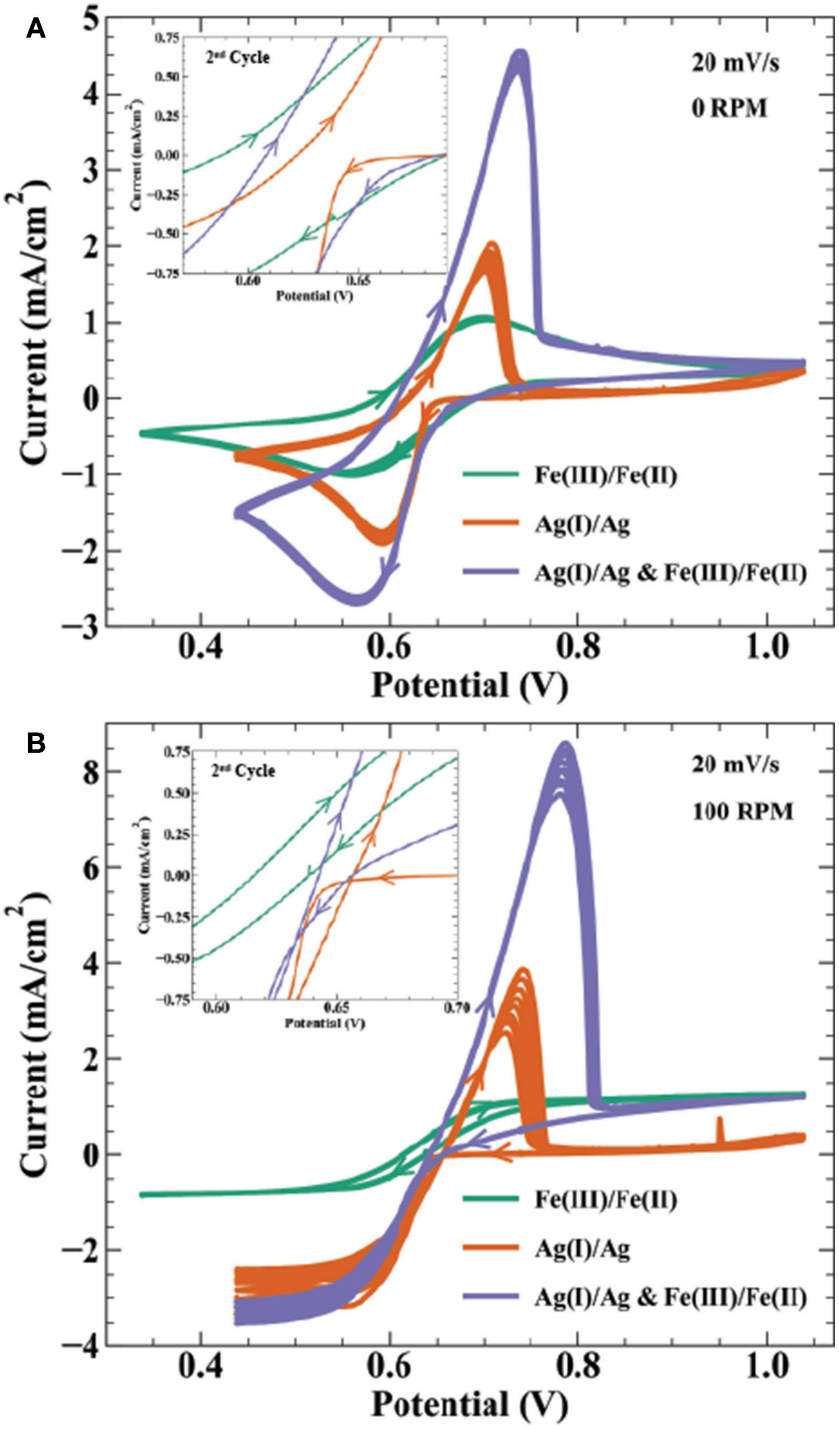
Cyclic voltammetry at 20 mV/s on a 0.5 cm Au electrode for 9 cycles at **(A)** 0 RPM and **(B)** 100 RPM in electrolyte containing 0.01 mol/L FeSO_4_ + 0.005 mol/L Fe_2_(SO_4_)_3_ (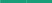), 0.01 mol/L AgNO_3_ (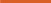), and 0.01 mol/L AgNO_3_ + 0.01 mol/L FeSO_4_ + 0.005 mol/L Fe_2_(SO_4_)_3_ (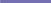). All solutions contain 0.1 mol/L K_2_SO_4_ as supporting salt. Inset figures highlight the voltammetric curves near zero current, showing the 2nd cycle for each CV.

Electroanalytical methods such as cyclic voltammetry help describe the thermodynamic and kinetic relationship between reversible electrodeposition systems and their bipolar oxidation couple. Figure 6 shows cyclic voltammetry relating the Ag^+^/Ag_(s)_ redox couple to the Fe^3+^/Fe^2+^ bipolar counter reaction described by Scheme 3. Specifically, CVs in Figure 6 are done for the following solutions: (1) 0.01 mol/L FeSO_4_ + 0.005 mol/L Fe_2_(SO_4_)_3_, (2) 0.01 mol/L AgNO_3_, and (3) 0.01 mol/L AgNO_3_ + 0.01 mol/L FeSO_4_ + 0.005 mol/L Fe_2_(SO_4_)_3_, with all three containing 0.1 mol/L K_2_SO_4_. At a rotation rate of 0 RPM (Figure 6A), solution (1) shows reduction of Fe^3+^ to Fe^2+^ with a peak current at 0.56 V and half peak potential of 0.635 V; oxidation of the reverse having a peak at 0.74 V and half peak potential of 0.645 V. The reversible Nernst potential estimated by CV of 0.64 V is in agreement with the measured open circuit potential of 0.63 V. For the silver only solution (2), deposition initiates after a small nucleation overpotential near 0.66 V reaching peak current at 0.63 V. On the return sweep, current crosses zero at 30 mV more positive than the iron solution, before exhibiting a characteristic metal stripping peak at 0.7 V.

Cyclic voltammetry in solution (3) with Ag(I), Fe(III), and Fe(II) ions results in a combination of features from voltammetry of the individual constituents. As potential is swept negative, peak current from combined ferric ion and silver ion reduction is at 0.57 V, with an estimated faradaic efficiency for silver reduction of 65% at this potential. Anodic stripping of silver in the return sweep appears more efficient than the Ag(I) solution. In solution (2), the total charge from the stripping wave is only 15% of the charge from the deposition wave, whereas solution (3) shows 86% of the total silver deposition charge in the stripping wave (estimated by assuming silver deposition efficiency is 65% throughout the combined Ag(I) and Fe(III) reduction wave). After silver is fully stripped, the i-V profile merges with that of solution (1) containing only ferric and ferrous ion components. Solution (3) voltammetry remains stable over 10 cycles despite the thermodynamic driving force for homogeneous silver reduction in solution.

Figure 6B shows the same solutions under forced hydrodynamics with a rotation rate of 100 RPM. The Fe(II)/Fe(III) redox couple exhibits strong stability over 10 cycles. The limiting currents for Fe(III) reduction and Fe(II) oxidation are −0.85 and 1.27 mA/cm^2^, respectively. Using the Levich equation and an assumed solution viscosity of 0.01 cm^2^/s, the calculated diffusivities for Fe(III) and Fe(II) are 2.9 × 10^−6^ cm^2^/s and 5.3 × 10^−6^ cm^2^/s, respectively. These values are comparable to previously reported diffusivities of 3–5.5 × 10^−6^ cm^2^/s for Fe(III) (Gil et al., 1996) and 1.1–5.7 × 10^−6^ cm^2^/s for Fe(II) (Andricacos et al., 1998; Hawthorne et al., 2014). Silver deposition in solution (2) shows variation in the transport limited current across 10 cycles, increasing in magnitude from 2.4 to −3.3 mA/cm^2^. The calculated diffusivity using the Levich equation for Ag(I) is 1.4–2.2 × 10^−5^ cm^2^/s, comparable to the literature value of 1.27 × 10^−5^ cm^2^/s (Okeefe et al., 1987). Peak anodic current in the stripping wave also decreases from 3.8 to 2.5 mA/cm^2^ from the first to last cycle, resulting in a stripping efficiency decrease from 34 to 20%. The combined Ag(I), Fe(II), and Fe(II) solution shows similar variation in the reduction and stripping waves as seen in the Ag(I) solution. A possible explanation for the low silver stripping efficiency in Figure 6 voltammetry is detachment of dendritic silver from the electrode surface prior to full oxidation of the deposited charge. The inset highlighting the region near zero current shows the Fe(III)/Fe(II) voltammetry crossing zero current between 0.62 and 0.64 V, consistent with the measured open circuit potential of 0.63 V. In the Ag(I) solution, the intersection of the return sweep with the forward sweep, often considered the reversible potential in deposition systems, occurs at 0.66 V. Cyclic voltammetry in Figure 6 and open-circuit voltage measurements indicate that the reversible potentials for the Fe(III)/Fe(II) and Ag(I)/Ag redox couples produce a bipolar electrolyte with Δ*E*_*BC*_ ≈ 30–40 mV. This thermodynamic cushion is sufficient for the temporally stable silver deposition observed in Figure 2c.

**Figure 7:**
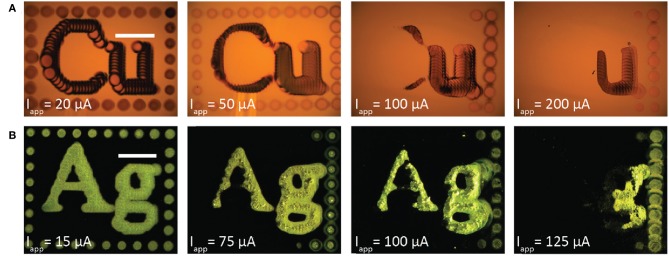
Optical micrographs of patterned metal deposited from the SBC with a 200 μm ID nozzle at 400 μL/min electrolyte flowrate, fly-height of 30 μm, 75 μm deposit spacing, 2 second dwell time per deposit, and the indicated applied currents for **(A)** copper from a 0.05 mol/L CuSO_4_ + 0.005 mol/L ascorbic acid electrolyte and **(B)** silver from a 0.01 mol/L AgNO_3_ + 0.01 mol/L FeSO_4_ + 0.005 mol/L Fe_2_(SO_4_)_3_ electrolyte. Copper patterns were imaged in bright field mode and silver images imaged in dark field mode.

Despite these efforts to design the thermodynamics of the bipolar redox couples for stable metal patterning, metal dissolution can still be kinetically activated if the substrate is polarized sufficiently. Figure 7 shows a series of experiments for bipolar copper electrodeposition (Figure 7A) and bipolar silver deposition (Figure 7B) performed at the indicated applied currents. Each pattern was deposited with a 200 μm ID nozzle at 400 μL/min flowrate, 30 μm fly-height, 75 μm deposit spacing, and 2 second dwell time per deposit. The copper electrolyte was 0.05 mol/L CuSO_4_ + 0.005 mol/L ascorbic acid and the silver electrolyte was 0.01 mol/L AgNO_3_ + 0.01 mol/L FeSO_4_ + 0.005 mol/L Fe_2_(SO_4_)_3_. Copper images were acquired in bright field imaging. Silver images, however, were acquired with dark field imaging because the coarse, large grained silver deposits scatter light and appeared dark in bright field (as in Figure 2c). For each metal, the pattern at the lowest current is fully retained on the gold substrate. However, as applied current increases more of the metal pattern is electrochemically etched from the surface; the SBC, beginning in the top left corner, rasters top-to-bottom and then left-to-right. Additional applied current creates a larger voltage drop beneath the nozzle, manifesting in increased substrate polarization and larger surface overpotentials for the bipolar reaction couple. The increase in surface overpotential exceeds the thermodynamic cushion designed into the metastable electrolytes, activating metal oxidation and resulting in pattern removal from the bipolar electrode.

**Figure 8:**
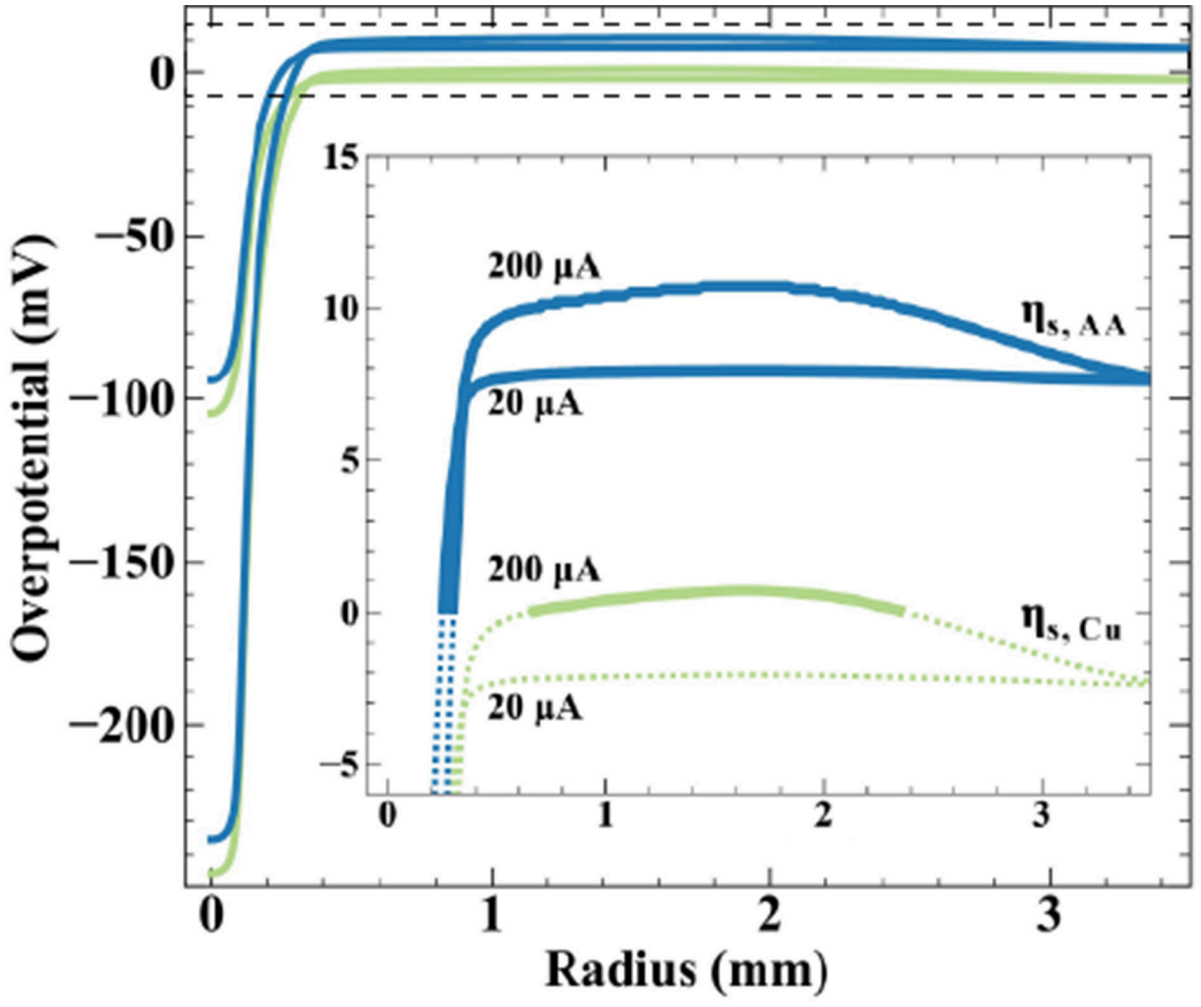
Simulated surface overpotentials for copper (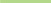) and ascorbic acid (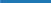) on the substrate for a 200 μm ID nozzle at a fly-height of 15 μm and the indicated applied currents. Inset emphasizes far-field oxidation region (denoted by the dashed box), with solid lines indicating oxidizing overpotentials and dashed lines indicating reducing overpotentials.

Finite element method simulations relate qualitative observations of Figure 7 to theoretical predictions regarding potential distribution on the bipolar electrode. Figure 8 shows simulated surface overpotentials for the copper (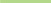) and ascorbic acid (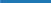) redox couples radially on the conductive substrate (zero is nozzle center) at the indicated applied currents. The surface overpotential is

(11) $$\begin{array}{l} \eta_{s.j}=V_{m}-\phi \left(r\right)-E_{j}^{eq} \end{array}$$

where the solution potential (ϕ) evaluated at the substrate surface is a function of radial position. Equilibrium potentials ($E_{j}^{eq}$) are listed in Scheme 2, producing a Δ*E*_*BC*_ = +10 mV, and the open-circuit mixed potential of the conductive substrate (*V*_*m*_) reflects the thermodynamics and kinetics of the reactions when net current and potential are equal to zero. Simulations use a fly-height of 15 μm and nozzle ID of 200 μm. The inset emphasizes overpotential in the far-field oxidation region, with solid lines indicating oxidizing overpotentials and dotted lines reducing overpotentials. At 20 μA, the peak cathodic overpotentials beneath the nozzle for copper and ascorbic acid are −105 and −95 mV, respectively. The most positive overpotentials appear in the far-field region of the substrate, equal to −2 mV for copper and 8 mV for ascorbic acid. The surface overpotentials of the bipolar redox couples maintain a separation of 10 mV (equal to the value for Δ*E*_*BC*_) across the substrate. The local copper overpotential (η_*s*.*Cu*_) is negative everywhere on the substrate, therefore, the only thermodynamically possible anodic chemistry is ascorbic acid oxidation. Despite the large cathodic overpotential for dehydroascorbic acid reduction, only moderate amounts of current are produced due to the trace amounts of DHAA present in solution, with a computed faradaic efficiency for copper reduction equal to 99.9%.

Increasing applied current to 200 μA generates greater substrate polarization, shifting peak cathodic overpotentials to −245 and −235 mV for copper and ascorbic acid, respectively. The magnitude of overpotential change in the far-field region is less substantial, a result of its much larger surface area, producing maximum overpotentials of 1 mV for copper and 11 mV for ascorbic acid. The substrate now exhibits a region with a positive value for η_*s*.*Cu*_ capable of driving copper oxidation. Simulations estimate a modest decrease in faradaic efficiency for ascorbic acid oxidation, from 100 to 92%. Increased substrate polarization as a result of higher applied currents kinetically activates metal oxidation, leading to the removal of material observed in Figure 7. Due to the uncertainty in measurements of the reversible potentials for the copper and ascorbic acid redox couples, the treatment of dilute DHAA in the electrolyte, and assumption of an ideal SBC nozzle geometry, these simulations are not intended to be quantitative predictors of experimental observations, but instead used as theoretical assessments of the underlying phenomena.

## Conclusions

The SBC provides a unique platform for electrochemical materials patterning on conducting substrates without direct electrical connections. Efficient bipolar patterning begins with effective electrolyte design, where understanding the kinetics of the desired bipolar reduction chemistry are critical for stable patterning. Metals with a large kinetic barrier to electrochemical oxidation are inherently stable on the bipolar electrode, permitting a wider range of redox couples suitable for the bipolar counter reaction as well as greater latitude in SBC operating conditions. Metals that are kinetically capable of dissolving must instead have stability built into the thermodynamic relationship between the desired reduction reaction and the bipolar counter reaction, limiting the redox couples available as bipolar counter reactions as well as the applied currents permitting spatiotemporal patterning. In addition to these bipolar design criteria, traditional electrolyte considerations such as pH balance and secondary parasitic reactions may impact deposit morphology and microstructure. Cyclic voltammetry measurements clarify experimental observations of secondary reactions during bipolar nickel deposition and support hypotheses for design guidelines of kinetically reversible electrodeposition systems. Finally, finite element method simulations indicate reduced bipolar electrode surface areas will dramatically impact BCE.

## Data Availability

The datasets generated for this study are available on request to the corresponding author.

## Author Contributions

TB conducted experiments and simulations. DS provided funding support. TB and DS provided input to the manuscript.

### Conflict of Interest Statement

The authors declare that the research was conducted in the absence of any commercial or financial relationships that could be construed as a potential conflict of interest.

## Appendix: Computational Model Details

A secondary current distribution is appropriate here because the concentration is substantially uniform, a result of high transport rates from the jetted electrolyte (limiting current densities can exceed 10 A/cm^2^), and thus concentration gradients can be neglected (Nelson and Schwartz, 2005). Potential distribution (ϕ) in the electrolyte is defined by Laplace's equation.

(A1) $$\begin{array}{l} \nabla^{2}\phi =0 \end{array}$$

The nozzle, housing walls, and electrolyte meniscus are treated as insulating boundary conditions,

(A2) $$\begin{array}{l} n\cdot \nabla \phi =0 \end{array}$$

where ***n*** is the unit normal vector pointing out of the computational domain. The feeder anode boundary condition at the microjet inlet is

(A3) $$\begin{array}{l} -\kappa n\cdot \nabla \phi =\frac{I_{app}}{A_{nozzle}} \end{array}$$

where κ is the electrolyte conductivity, *A*_nozzle_ is the area of the nozzle where current is applied and *I*_app_ is the total applied current to the system. The feeder cathode boundary condition is

(A4) $$\begin{array}{l} -\kappa \text{n}\cdot \nabla \phi =\frac{-I_{app}}{A_{nozzle}} \end{array}$$

with the area, *A*_cathode_, equal to the area of the platinum outer ring cathode.

The most general form for the bipolar reaction rates at the conductive substrate is given by a modified Butler-Volmer kinetic approximation

(A5) $$\begin{array}{l} i_{j}=i_{o,j}\left[f_{j}e^{\left(\frac{\alpha_{a,j}n_{j}F}{RT}\eta_{s,j}\right)}-g_{j}e^{\left(\frac{-\left(1-\alpha_{a,j}\right)n_{j}F}{RT}\eta_{s,j}\right)}\right] \end{array}$$

where *i*_o,j_ is the exchange current density for reaction *j, n*_j_ is the number of electrons transferred for reaction *j* and α_a,j_ is the transfer coefficient for the anodic branch of reaction *j*. The parameter *T* is the temperature of the system (298 K), *F* is Faraday's constant, and *R* is the ideal gas constant. The surface overpotential in Equation A.5 is defined by Equation 11. The functions *f*_j_ and *g*_j_ modify the Butler-Volmer equation to account for the reversibility of the respective branches. For example, the general Butler-Volmer equation with reversible kinetics, such as for copper, has functions *f*_j_ and *g*_j_ both equal to 1. In the case of an irreversible chemistry such as nickel, a Tafel approximation for reduction is produced when *f*_j_ = 0 and *g*_j_ = 1. The total current at the substrate is the sum of the individual partial currents:

(A6) $$\begin{array}{l} i=\sum i_{j}. \end{array}$$

To remain charge neutral, the conductive substrate requires equal magnitudes of reduction and oxidation reactions, which is expressed by the integral constraint over the substrate area

(A7) $$\begin{array}{l} I_{e,net}=2\pi \int_{o}^{r_{sub}}irdr=\sum I_{e,red}+\sum I_{e,ox}=0 \end{array}$$

where *i* is defined by Equations A.5-A.6 and *I*_e,net_ is the net electronic current passing through the conductive substrate, equaling zero when integrated over the entire substrate. The currents *I*_e,red_ and *I*_e,ox_ are contributions from any bipolar reactions occurring on the substrate. The location along the radial axis where the partial current density for each reaction equals zero is defined as a bipolar cross-over point (*BPX*), with each bipolar couple yielding a *BPX*. Integrating the current density on the substrate from the nozzle center (*r = o*) to the BPX yields *I*_e,red_ to calculate *BCE* in Equation 1.

For simulations of near-field copper reduction in Scheme 2, the treatment of the small amounts of dehydroascorbic acid reduction by setting *g*_AA_ = (1-*i/i*_o,AA_) with *f*_AA_, *g*_Cu_, and *f*_Cu_ = 1 results in a boundary condition for total current density on the bipolar substrate as

(A8) $$\begin{array}{l} -\kappa n\cdot \nabla \phi =i=i_{o,AA}e^{\left(\frac{\alpha_{a,AA}n}{RT}\eta_{s,AA}\right)}\\ \;-i_{o,AA}\left[1-\frac{i}{i_{o,AA}}\right]e^{\left(-\frac{\left(1{-\alpha }_{a,AA}\right)n}{RT}\eta_{s,AA}\right)}\\ \;-i_{o,Cu}[e^{\left(\frac{\alpha_{a,Cu}n}{RT}\eta_{s,Cu}\right)}-e^{\left(-\frac{\left(1-\alpha_{a,Cu}\right)n}{RT}\eta_{s,Cu}\right)}] \end{array}$$

where *n* = 2 since both electrons transfer two reactions and other model parameters are listed in Table 2.
